# Moving from ‘personal communication’ to ‘available online at’: preprint servers enhance the timeliness of scientific exchange

**DOI:** 10.1186/s13034-019-0301-4

**Published:** 2019-10-31

**Authors:** Daniel Poremski, Bruno Falissard, Jörg Fegert, Andreas Witt, Anna E. Ordóñez, Andrés Martin, Daniel Shuen Sheng Fung

**Affiliations:** 1 Health Intelligence Unit, Institute of Mental Health, Singapore, Singapore; 2 CESP INSERM U1018, Université Paris-Saclay, AP-HP, Paris, France; 3 Department of Child and Adolescent Psychiatry and Psychotherapy, University of Ulm, Ulm, Germany; 4 Office of Clinical Research, National Institute of Mental Health, Bethesda, MD USA; 5 Child Study Center, Yale School of Medicine, New Haven, CT USA; 6 Yong Loo Lin School of Medicine and DUKE NUS Medical School, National University of Singapore, Singapore, Singapore; 7 Lee Kong Chian School of Medicine, Nanyang Technological University, Singapore, Singapore

## Context: preprints in health care

A preprint is a scientific manuscript uploaded by its authors into a public server prior to peer review or publication. The preprint contains the complete information of a scientific article and can be described as an ‘interim research product’ because it has not yet gone through the back-and-forth edits that typically occur during peer review. Rather, after a brief quality-control inspection by the preprint server to ensure that the work is scientific in nature and meets ethical standards, the manuscript is posted promptly (usually within a day or two) and can be viewed online for free by anyone. This allows for authors to receive prompt feedback from a far larger community of colleagues than the two or three experts who might typically review their manuscript. It also increases the visibility and speed with which research findings are disseminated and can help to counterbalance the effects of publication bias. Work posted as a preprint is frequently the same exact manuscript being submitted to a traditional, peer-reviewed journal, often simultaneously. In this way, preprints (which are efficient and rapid, but not validated through peer-review), can work in tandem with journal publications (which are slow, but provide validation through peer-review) as a communication system seeking to improve scientific research [1].

Preprints have been standard fare in physics and other hard sciences for the past three decades. In fact, publication in traditional venues is at times viewed as a late formality in these disciplines, where the bulk of the intellectual exchange can happen long before publication, through commentary around the preprint. Much of that exchange happens through email and social media, rather than through the preprint server itself, which is in essence but a virtual repository providing a public ‘time stamp’ to the work. Compared to the preprints’ immediacy in jump-starting dialogue, the traditional peer-reviewed approach can feel painfully slow to scientists eager to get their work out into the public discourse arena.

Compared to physics, the biological sciences have been slower on the preprint uptake, but a groundswell began in 2013, when the Cold Spring Harbour Laboratories launched bioRxiv. A further boost came in early 2017, when funding agencies encouraged applicants to include their preprint works in grant proposals. This invitation was supported both in the UK by the Medical Research Council [2] and the Wellcome Fund [3], and in the US by the National Institutes of Health [4] and the Howard Hughes Medical Institute [5]. The first repository specific for the health sciences, medR*x*iv, was started in 2019 [6, 7]. Concerns over the potential harm of faulty clinical studies, balanced against the possibility of making critical information available as soon as possible, may explain the lag in medical preprint servers, compared to those in other fields. The Table summarizes a representative sampling of preprint servers relevant to child and adolescent mental health (Table 1).

**Table 1:** Selected pre-print archives relevant to child and adolescent mental health

Archive	Homepage	Disciplines	Inception	Formats for submission	Formats published	Owned/operated by
bioRxiv	http://biorxiv.org	Biology	2013	PDF, Word	PDF	Cold Spring Harbor Laboratory
IACAPAP ArXiv	http://arxiv-iacapap.org/	Child psychiatry	2015	PDF	PDF	IACAPAP
OSF preprints medRxiv	https://osf.io/preprints/ https://www.medrxiv.org	All Health sciences	2016 2019	Any Any	Any Any	Open Science Framework Cold Spring Harbor Laboratory, BMJ, Yale
PeerJ preprints	https://peerj.com/preprints-search/	Biological Sciences, Medical Sciences, Health Sciences and Computer Sciences	2013	PDF	PDF and HTML	PeerJ
Preprints.org	http://www.preprints.org	All	2016	Word, LaTeX	PDF	MDPI
PsyArXiv	https://psyarxiv.com/	Psychology	2016	Any	Any	Open Science Framework
ResearchGate	https://www.researchgate.net	All	2008	Any	Any	ResearchGate

All archives use DOI (digital object identifier) as unique identifiers, and CC-BY (Creative Commons) for licensing purposes

Despite the availability of a number of preprint services, uptake remains low and uneven: for example, only 1.3% (1200) of the 93,000 papers added to PubMed in August of 2017 had been deposited as preprints [8]. This rate is likely to increase over time, particularly in light of emerging evidence that articles with a preprint received higher Altmetric attention scores and more citations than articles without a preprint [9]. Although preprints were not well cited in that study, 18% had Altmetric scores in the top 5th percentile, and 48% were estimated to reach peer-reviewed publication within 1 year. For context, Altmetric scores provide a weighted count of all of the online attention for an individual research product, including mentions in the ‘gray literature’ of public policy documents, references in Wikipedia, the mainstream news, social networks, blogs and more.

## Perspectives on preprints

The researcher’s *raison d’être* is to answer questions worthy of being asked, design methodologically sound ways of answering those questions and sharing the answers with those who may benefit from them. At each step of the process, the researcher depends immensely on peers, both present and past. While some have pointed out that this idyllic set of processes is not reflected in actual practice [10], it remains important that academics aspire to these ambitions.

There are several considerations that can inform a prospective author’s decision to upload a manuscript into a preprint archive:

### Location

With the rapid expansion of journals in the past three decades [11], particularly of open-access journals, it is hard to imagine that any particular niche of academia is unserved. However, with this immense diversification comes a challenge of choice and a diffusion of responsibility. Highly specialized journals may not be receptive to publications that fall slightly outside their areas of focus, resulting in the rejection of submissions not because of quality but because of an imperfect fit. This diversification and increased selectivity may mean that authors struggle to find an optimal place for their article, especially problematic for early career researchers who are still developing their academic identity and expertise. Although preprint archives can also be selective, they may be less so than specialized journals. Therefore, they may serve as a convenient temporary space for a manuscript that has yet to find a suitable journal, not by fault of its quality, but by virtue of its specialization.

### Timeliness

Journals have sought to reduce as much as possible the lag between submission and dissemination, providing rapid editorial decisions and early access to proofs and electronic copies. But the review and publication process does take time, and some have argued for time-to-publication bias in the selection of articles [12]. Offering a timely avenue to public distribution is one of the main selling points of so-called ‘predatory journals’. Definition of that term remains somewhat nebulous, but in general denotes a periodical seeking to publish articles for a fee rather than for scientific merit [13]. But it should be noted that timeliness is likewise a virtue of preprint archives. Therefore, if time is of the essence and timeliness the main reason an author would consider a fee-for-service journal that boasts blistering processing times (at a potential cost of journal reputation), it may be more reasonable to seek to register the preprint while submitting to a suitable journal with its longer processing time. Indeed, moving from referring to an unpublished manuscripts as ‘personal communication’ to the more robust ‘available online at’ represents not just a less eyebrow-raising designation. It provides a direct way to access the material and to make it available for dialogue, critique and further refinement en route to publication. In this way, the preprint ‘developmental stage’ of a scientific manuscript represents an opportunity for the maturing of its scientific content, and allows investigators to make their material widely available during the often lengthy path through ‘in press’ and into final appearance in print and pixel.

### Predatory journals

Predatory journals have taken advantage of the growing popularity and demand for open-access publication. This puts academia at risk because it offers a compromised avenue to the literature. In the journal ecosystem and its overabundance of titles, an ill-informed consumer might be forgiven for massing reputable and disreputable journals together, taking information provided at face value and trusting the peer review process. One could argue that the ecological niche populated by predatory journals would not be as viable if authors were able to secure a legitimate place in the literature for their unreviewed work. Expanding free archiving services may help undercut the predatory journal business model.

### Cultural and linguistic diversity

A special area of medical and psychiatric research, particularly in child and adolescent psychiatry, is that cultural context can strongly influence the clinical practices, and even the effectiveness of treatment. This is especially true when considering non-pharmacological treatments. As most peer-reviewed scientific journals only consider papers written in English [14, 15], editors can also be reluctant to accept publications dealing with strong cultural or linguistic specificities. This is based on the idea that cultural specificity limits generalisability. Over time, there is a significant risk that effective treatments developed in remote, linguistically underrepresented, or impoverished [16, 17] cultures will not be recorded. Preprint archives provide a unique opportunity to preserve and expand cultural diversity in etiology and therapeutics, including of culture-bound conditions.

### Access

Access is the main issue that justifies devoting resources to preprint archives. Why bother at all? These archiving services are not heavily accessed and are not currently indexed on the main sources of academic literature. While Digital Object Identifiers (DOIs) are assigned to each archived manuscript, currently they are only retrievable by means of actively informed search strategies. Therefore, while a manuscript may be archived, it may not be as widely accessed as articles that appear in peer-reviewed indexed journals. But as the practice becomes more widely adopted—as has been the case in many other branches of science– we can anticipate that it will become as natural to search a centralized preprint archive as it is to search multiple sources of peer-reviewed articles today. Additionally, searching preprint archives will offer researchers a glimpse into ongoing research, something not easily accomplished without the proper connections. It therefore benefits researchers to adopt the habit of searching preprint archives, as they offer an idea of what is to come and consequently what remains unanswered.

### Trustworthiness

The idea of registering intellectual property with a publicly accessible server is appealing, but without the scrutiny of an independent third party the degree of trustworthiness of the content may be called into question. Those supporting the practice can justifiably point to the number of articles appearing as preprints that then go on to be published in peer-reviewed journals: about 55% after 24 months [18]. This rate of transition indicates that the use of preprint services, at least currently, is not a repository of articles that are not up to the same standard of peer-reviewed articles, but simply that they have been placed in the registry to be disseminated as expeditiously as possible. It is possible that as the popularity of preprint archives grows the number of unscrupulous researchers seeking to disseminate unworthy content will grow as well. But if disciplines adopt preprint practices and integrate them into their publishing process, we can anticipate that the sophistication of the vetting process will improve, reducing the risks associated with simply accepting submissions with a low degree of scrutiny.

## Preprints in Child and Adolescent Mental Health

Biomedical journal publishers are warming to the idea of preprint archives [19], which should assuage worries about preprints compromising the potential for future publications. Despite the recent expansion in preprint interest in biomedicine, psychiatric and psychological sciences have not yet fully adopted the practice. Some have justifiably tempered their enthusiasm for adopting preprints in the medical fields, notably by citing the risks involved when sharing unreviewed medical advancements [20]. These risks are noteworthy, and judgement may be necessary to vet the inclusion of potentially dangerous content. However, establishing discipline-specific archives can help reduce the risk, as discipline-specific curators can provide more reliable vetting of submissions.

The International Association of Child and Adolescent Psychiatry and Allied Professions (IACAPAP) has developed a preprint archive specific to child and adolescent psychiatry (http://arxiv-iacapap.org/). This repository gives child and adolescent mental health professionals the opportunity to upload clinical or research documents in their own language (with an abstract in English) that becomes freely available online. The quality of the articles is assured by formal approval of the national child and adolescent mental health organization to which the authors belong, and/or by IACAPAP. The IACAPAP ArXiv is one of several preprint services that investigators may consider when uploading their work for circulation and dissemination. Most peer-reviewed journals, though not all, have policies specifically allowing the use of preprint servers prior to submission. In some instances, journal submissions from the preprint server can even happen directly. Authors are advised to check with the specific journal to which they intend to submit to before uploading their work to a preprint repository.

*CAPMH* and its editorial team certainly welcome, and indeed encourage, the use of preprint archives. We believe that any avenue that allows a researcher easy and timely access to sharing and disseminating findings should be encouraged and welcomed, as early access helps researchers better fulfil their *raison d’être*.
